# Stimulation of Nuclear Factor (Erythroid-Derived 2)-like 2 Signaling by Nucleus Targeted Irradiation with Proton Microbeam

**DOI:** 10.3390/biology12030419

**Published:** 2023-03-09

**Authors:** Jun Wang, Masakazu Oikawa, Teruaki Konishi

**Affiliations:** 1 The Center for Ion Beam Bioengineering and Green Agriculture, Hefei Institute of Physical Science, Chinese Academy of Sciences, Hefei 230031, China; 2 Single Cell Radiation Biology Team, Quantum Life and Medical Science Directorate, National Institutes for Quantum Science and Technology, 4-9-1 Anagawa, Chiba 263-8555, Japan; 3 Electrostatic Accelerator Operation Section, Quantum Life and Medical Science Directorate, National Institutes for Quantum Science and Technology, 4-9-1 Anagawa, Chiba 263-8555, Japan

**Keywords:** microbeam, NRF2, nucleus targeted irradiation, ATM, ERK 1/2

## Abstract

**Simple Summary:**

With the advancement in microbeam cell irradiation technologies, researchers can now investigate subcellular region irradiation-induced biological responses as well as whether and how they contribute to the overall radiation induced biological effects of individual cells and the cell population. It will be of great value not only in expanding our understanding of the mechanisms of ionizing interaction with cells, but also in the studies of radiation protection and radiotherapy. Radiation-induced biological effects are commonly associated with an increase in reactive free radicals. Nuclear factor erythroid 2-related factor 2 (NRF2) is a master regulator of cellular oxidative stress. We investigated the response of NRF2 signaling to nucleus targeted irradiation using the Single Particle Irradiation System to Cell (SPICE-QST microbeam) facility and evaluated the similarities and differences between irradiating the cytoplasm or nucleus in the stimulation of NRF2 signaling.

**Abstract:**

Nuclear factor (erythroid-derived 2)-like 2 (NRF2), well-known as a master antioxidative response regulator in mammalian cells, is considered as a potential target for radiation protection and cancer therapy sensitization. We examined the response of NRF2 signaling in normal human lung fibroblast WI-38 cells to nucleus targeted irradiation by 3.4 MeV proton microbeam. Nucleus targeted irradiation stimulated the nucleus accumulation of NRF2 and the expression of its target gene, heme oxygenase 1 (HO-1). The nucleus accumulation of NRF2 increased from 3 h to 12 h post 500 proton irradiation. In the 500 protons range, higher number of protons resulted in increased NRF2 nucleus accumulation. Activating NRF2 with *tert*-butylhydroquinone reduced DNA double-strand break (DSB) formation in nucleus targeted irradiation by 15%. Moreover, ATM phosphorylation was found in nucleus targeted irradiation. Inhibiting ATM with ku55933 prevented NRF2 nucleus accumulation. Furthermore, nucleus targeted irradiation activated ERK 1/2, and ROS-ERK 1/2 signaling regulated NRF2 nucleus accumulation. Taken together, NRF2 signaling was activated by nucleus targeted irradiation and mitigated DNA DSB. The discovery of ATM and ERK 1/2 as upstream regulators of NRF2 signaling in nucleus targeted cells revealed new information regarding radiation protection.

## 1. Introduction

Microbeam irradiation with micron-sized beams is a powerful tool for investigating subcellular region targeted irradiation-induced biological effects [1,2], radiation-induced bystander [3] and rescue effects [4], radiation-induced adaptive response [5], and individual cell irradiation-induced cell–cell communication [4]. For example, Hei et al. [6] and Wu et al. [7] discovered the mutagenic effects of nucleus and cytoplasm targeted irradiation, elucidated the mutagenic characteristics, and clarified the role of reactive oxygen species in cytoplasm targeted irradiation-induced gene mutations using an alpha particle microbeam. Shao et al. demonstrated that cytoplasm targeted by helium ions (^3^He^2+^) resulted in radiation-induced bystander effects (RIBE) and identified nitrogen oxide as an important signal factor [8]. The team from the National Institute of Radiological Sciences of Japan discovered that RIBE and rescue effects co-existed in a mixed culture system containing A549 cells and WI-38 cells using the SPICE-QST proton microbeam [9,10]. They also demonstrated that cytoplasm irradiation motivated adaptive responses [5] and enhanced DNA double-strand break (DSB) repair [11]. Matsumoto et al. compared the micronucleus formation in bystander cells and neighboring cells targeted by carbon, neon, or argon ions using a heavy-ion microbeam at Takasaki Ion accelerators for Advanced Radiation Application (TIARA) [12], and found a dose dependent tendency [13]. Autsavapromporn et al. discovered the beam quality and irradiation dose dependency in micronucleus formation in bystander cells as well as the different contributions of gap-junction intercellular communication in the induction of micronucleus formation in bystander cells and neighboring cells targeted by various types of microbeam facilities [14].

An elevated production of reactive free radicals is involved in radiation-induced DNA damage [15]. Nuclear factor erythroid 2-related factor 2 (NRF2) is an important regulator of cellular oxidative stress [16]. Under homeostasis, NRF2 is sequestered in the cytoplasm and degraded through ubiquitination. When a cell is subjected to increased oxidative stress, NRF2 dissociates from its inhibitory proteins and translocates from the cytoplasm to the nucleus, where it transcriptionally regulates the expression of detoxification enzymes containing antioxidant responsive elements upstream of their promoters, thereby reducing cellular oxidative stress. NRF2 targets include heme oxygenase 1 (HO-1), thioredoxin, and gamma glutamylcysteine synthetase. Furthermore, the activation of NRF2 and its targets will confer the resistance of cells to DNA damage inducers including ionizing irradiation. For example, You et al. [17] discovered that the actomyosin cytoskeleton component myosin heavy chain 9 (MYH9) facilitated radio-resistance in head and neck cancer call by activating NRF2 and modulating cellular ROS levels. Matsuoka et al. [18] discovered that increased NRF2 dependent glycolysis and glutathione synthesis conferred oral squamous cell carcinoma radio-resistance. Furthermore, Kim et al. [19] found that 53BP1 was a direct target of NRF2. The chemical inducer of NRF2 protected colonic epithelial cells from ionizing irradiation. Given the fact that NRF2 is closely involved in the regulation of radiation induced biological effects, we verified that cytoplasm targeted irradiation increased the mitochondrial superoxide level, which resulted in the activation of the NRF2 signal and the alleviation of cytoplasmic irradiation-induced DSB levels in human lung fibroblast WI-38 cells. We also found that the mitochondrial morphology changes in the cytoplasm targeted cells contributed to NRF2 nucleus accumulation [20]. In this study, we investigated whether NRF2 signaling was activated and mitigated DNA damage in the nucleus targeted cells by utilizing a SPICE-QST proton microbeam facility.

## 2. Materials and Methods

### 2.1. Cell Culture

The RIKEN Bioresource Center provided the normal human lung fibroblast WI-38 used in this study (Ibaraki, Japan). WI-38 cells were cultured in D-MEM (Wako Pure Chemical Industries Ltd., Osaka, Japan) supplemented with 10% fetal bovine serum (ThermoFisher Scientific, Tokyo, Japan) plus 100 µg/mL streptomycin and 100 U/mL penicillin (ThermoFisher Scientific, Tokyo, Japan) at 37 °C in a humidified atmosphere of 5% CO_2_ at 37 °C.

### 2.2. Antibodies and Chemicals

Antibodies used were anti-NRF2 (Abcam Cambridge, UK), anti-phospho-histone H_2_A.X (Ser139), anti-phospho-ATM (Ser1981) (Merck KGaA, Darmstadt, Germany), anti phospho-ERK 1/2 (Cell Signaling Technology, Danvers, MA, USA), and anti-heme oxygenase 1 (HO-1) (Proteintech, Rosemont, IL, USA). ThermoFisher Scientific provided the Alexa Fluor-488 conjugated Goat Anti Rabbit and Alexa Fluor-555 conjugated Goat Anti Mouse secondary antibodies. The MEK 1/2 chemical inhibitor U0126 was purchased from Promega (Beijing, China). The ATM chemical inhibitor ku55933 was from Santa Cruz (Dallas, TX, USA). The chemical inducer of NRF2 *tert*-butylhydroquinone (*t*BHQ) was purchased from Sigma (Merck KGaA, Darmstadt, Germany). Hoechst 33342 and hydroethidine were purchased from ThermoFisher Scientific.

### 2.3. Nucleus Targeted Irradiation with Proton Microbeam

Cell irradiation was performed on the Single Particle Irradiation system to Cell (SPICE-QST microbeam) facility at the National Institutes for Quantum Science and Technology (QST), Chiba, Japan [9]. The physical characteristics of the SPICE-QST, cell culture techniques, and irradiation settings are detailed elsewhere [9]. Briefly, SPICE-QST provides a microbeam of 3.4-MeV protons with an approximately 2-μm beam diameter and a precise number of protons to the targeted area. The bottom of the specially designed microbeam cell culture dish was sealed with Chemplex Industries 6 µm thick polypropylene film (Palm City, FL, USA). After sterilization, the microbeam dishes were treated for 30 min at 37 °C with 1 µg/mL fibronectin dissolved in PBS. Then, the dishes were washed with phosphate buffered saline (PBS) and ready for use. A total of 1 mL of the WI-38 cell suspension was plated into the dish (cell density was approximately 15,000 cells/mL) and cultured for 48 h. Cell culture medium was replaced 0.5 h before irradiation with complete medium containing 1 µg/mL Hoechst 33342 (Dojindo Laboratories, Tokyo, Japan) for localization of the cellular nucleus in the following microbeam irradiation. Just before irradiation, the medium was drained off from the microbeam dish. A 6-µm thick polypropylene film was used to cover the cells during the microbeam irradiation process to prevent dehydration.

The dish was placed on the X–Y voice-coil motorized stage of the SPICE-QST microscopic system to obtain fluorescence images of the nuclei in a 3.28 × 3.28 mm^2^ area. Each nucleus was considered elliptical to calculate the central coordinate of the nucleus. For each dish, all procedures including image capture, X–Y coordinate calculation, and irradiation, were completed in less than 10 min. The polypropylene film used to cover the cells was removed after microbeam irradiation. Cell culture medium was added to the dish, and the cells were incubated until analysis.

### 2.4. Immunofluorescence Staining

For the immunofluorescence detection of NRF2 and phospho-ERK 1/2, cells were fixed with −20 °C methanol for 10 min. The dishes were then dried on a clean bench before being blocked for 2 h at room temperature with blocking buffer (PBS buffer, 0.1% Triton X-100, and 1% BSA). The primary antibody, diluted in blocking buffer, was added into the dish and the samples were incubated at 4 °C overnight. After three washes with PBST (PBS buffer containing 0.1% Triton X-100), the samples were incubated for two hours at room temperature in a dark box with gentle shaking with the corresponding Alexa Fluor conjugated secondary antibody diluted in blocking buffer. After washing with PBST, the cell nucleus was stained with 1 ug/mL Hoechst 33342 for 10 min. The images were captured on a SPICE offline microscope system equipped with a 40× water immersion objective lens (LUMPLFLN-W 40 × W, NA:0.8, Olympus Co, Tokyo, Japan), 1.6× intermediate lens (U-CA, Olympus Co, Tokyo, Japan), and a C-MOS camera (ORCA-ER FLASH4.0, Hamamatsu Photonics K.K., Hamamatsu, Japan). The image size was 207 μm × 207 μm (1024 × 1024 pixel, 0.0408 μm^2^ per pixel). Other proteins were detected by immunofluorescence after the cells were fixed with 4% paraformaldehyde for 20 min and then treated with PBST at room temperature for 30 min. Other steps were the same as those used for the immunofluorescence detection of NRF2. NIH ImageJ software was used to analyze the images (Bethesda, MD, USA). Briefly, we first captured the Hoechst33342 fluorescence of each region in the cell dish with the same exposure time, and then the fluorescent images of each region according to the fluorescent probe used. The parameters such as exposure time and intensity of the light source were kept the same for the fluorescent probe. For NRF2, phos-H2A.X, we first used the corresponding Hoechst33342 fluorescence image to determine the nucleus area of all cells in the image as our region of interest (ROI). These ROIs will be automatically recorded by ImageJ. Then, the averaged fluorescence intensities of the Alexa Fluor and the sizes of each ROI were measured by ImageJ and recorded for further analysis. For HO-1 and phos-ERK 1/2, we first used the fluorescence image of Alexa Fluor to set up the cell boundary by adjusting the threshold. The outlines of cells were recorded as ROIs. Then, the fluorescence intensities of each ROI were measured for analysis. Overall, the measurement of the fluorescence intensities was carried out region by region. In each region, the fluorescence intensities of each ROI were measured. These fluorescence intensities were collected and analyzed.

### 2.5. Measurement of Intracellular ROS

A final concentration of 2 μmol/L hydroethidine was added to the cell culture medium 30 min before microbeam irradiation to measure the superoxide. Sixty minutes after radiation, the cells were gently washed with warm PBS, and then the fluorescence was captured under the SPICE offline microscope system. The fluorescence intensity was analyzed with NIH ImageJ software. 

### 2.6. Statistical Analysis

For each sample, at least 150 cells were examined. Statistical data were represented as the mean ± standard error from at the least three independent experiments. The Student’s t test was used to determine the statistical significance of differences between two groups. *p* value < 0.05 between groups was considered to be of statistical significance.

## 3. Results

### 3.1. Nucleus Targeted Irradiation Promotes the Nucleus Accumulation of NRF2

**Figure 1:**
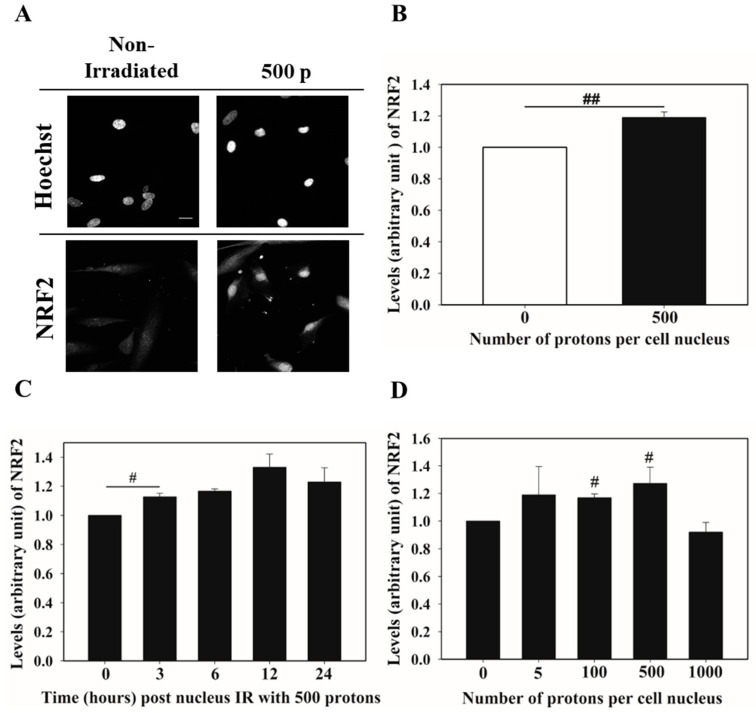
A total of 500 protons were irradiated at the nucleus of WI-38 cells. Twenty-four hours later, immunofluorescence staining revealed the increase in NRF2 in the nuclei. Scale bar = 30 µm (**A**,**B**). The nucleus of the WI-38 cells was targeted by 500 protons and then incubated. Immunofluorescence staining was used to identify the levels of NRF2 in the nuclei at various time points after irradiation (**C**). The nucleus of the WI-38 cells was targeted by different amounts of protons. Twelve hours later, the nucleus accumulation of NRF2 was detected by immunofluorescence staining (**D**). # *p* value < 0.05. ## *p* value < 0.01.

**Figure 2:**
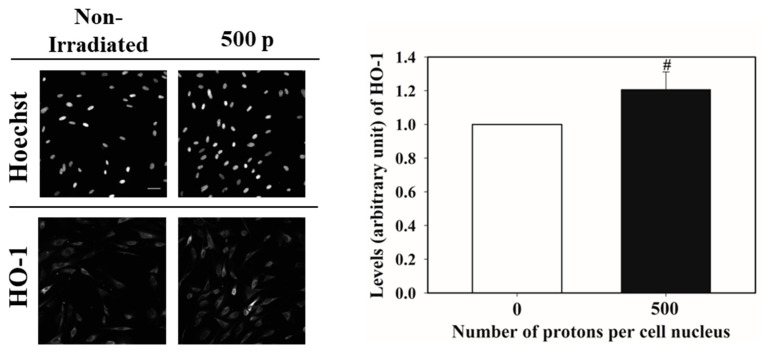
The 500 protons were irradiated at the nucleus of the WI-38 cells. Cellular HO-1 levels were detected by immunofluorescence staining 12 h later. Scale bar = 50 µm. # *p* value < 0.05.

The WI-38 cell nucleus was targeted with 500 protons. Twenty-four hours later, the nucleus accumulation of NRF2 was analyzed by immunofluorescence staining (Figure 1A). The nuclear level of NRF2 in the targeted cells increased around 20% compared to the non-irradiated cells, according to the fluorescence intensity of NRF2 (Figure 1B). The accumulation of NRF2 in the nucleus was then detected at the 0, 3, 6, 12, and 24 h time points following 500 protons of nucleus targeted radiation. As shown in Figure 1C, nucleus accumulation of NRF2 was observed 3 h after irradiation and reached a maximum 12 h later, to a level around 30% higher than that in the non-irradiated cells. We next detected the radiation dose dependency of NRF2 nucleus accumulation. The WI-38 cell nuclei were targeted with 0, 5, 100, 500, and 1000 protons and then incubated for 12 h before NRF2 immunofluorescence staining. At the tested doses, 100 and 500 protons significantly increased the NRF2 nucleus accumulation by factors of 1.20 and 1.30, respectively. The detected NRF2 level was lower than that in the non-irradiated cells when the dose was increased to 1000 protons (Figure 1D). We further detected the levels of heme oxygenase-1 (HO-1), one of the typical downstream targets of NRF2, in the WI-38 cells after nucleus irradiation. Figure 2 shows that 12 h after 500 proton irradiation, the cellular HO-1 levels increased by around 21%. These results indicate that nucleus targeted irradiation stimulated the NRF2 antioxidative response in WI-38 cells.

### 3.2. Activation of NRF2 Alleviates Nucleus Irradiation Induced DNA Double-Strand Breaks

**Figure 3:**
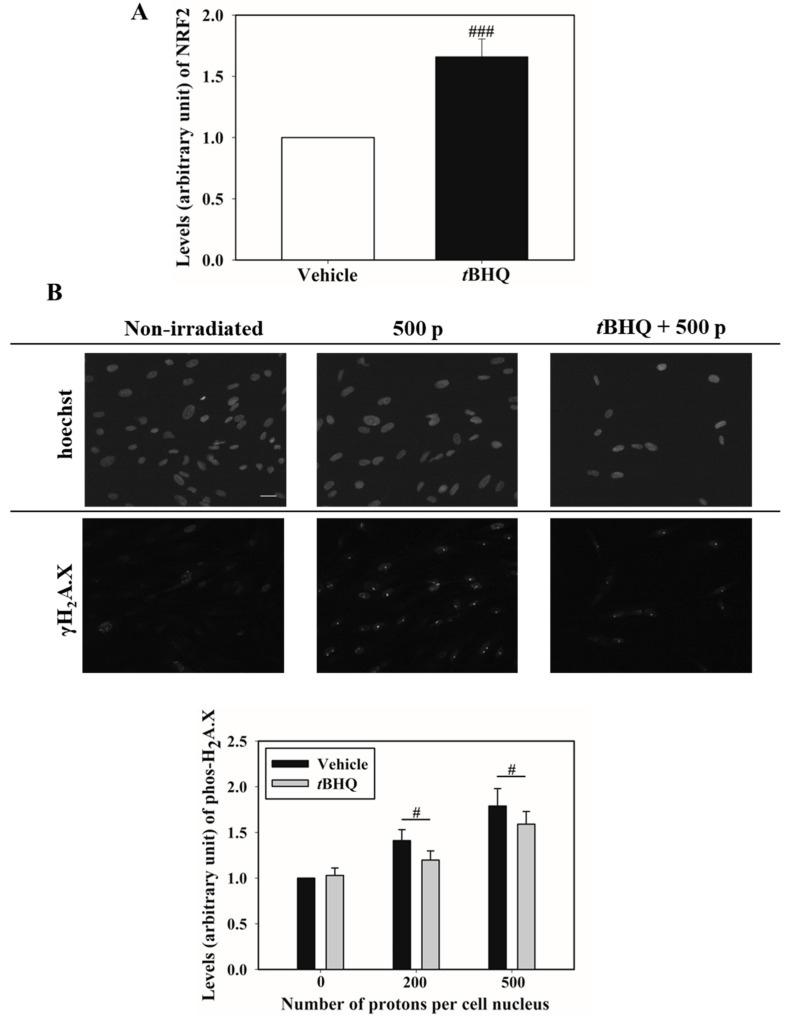
WI-38 cells were treated with 15 μmol/L of tBHQ or the vehicle for 16 h. Then, the nucleus accumulation of NRF2 was identified by immunofluorescence staining (**A**). WI-38 cells were treated with 15 μmol/L of tBHQ or the vehicle for 16 h. Then, the cell nucleus was targeted with 200 or 500 protons. Three hours later, the levels of γ-H_2_A.X were detected by immunofluorescence staining. Scale bar = 30 µm (**B**). # *p* value < 0.05. ### *p* value < 0.001.

Next, we investigated whether activating NRF2 reduced the nucleus targeted irradiation-induced DNA DSB in the WI-38 cells. *t*BHQ is a chemical activator of NRF2. Pretreatment of WI-38 cells with 15 μmol/L *t*BHQ for 16 h significantly increased the NRF2 nucleus accumulation (Figure 3A). Then, the cell nucleus was exposed to 200 or 500 protons. Three hours later, the levels of the DSB marker γH_2_A.X were detected by immunofluorescence staining. Figure 3B shows that *t*BHQ pretreatment resulted in a 15% decrease in the γH_2_A.X levels in the 200 proton irradiated cells when compared to the irradiated cells pretreated with the vehicle. Moreover, in the 500 proton irradiated cells, similar tendency was observed. This finding indicated that activating NRF2 reduced the level of DNA damage caused by nucleus targeted irradiation.

### 3.3. ATM Activation Is Involved in Stimulating NRF2 Nucleus Accumulation

**Figure 4:**
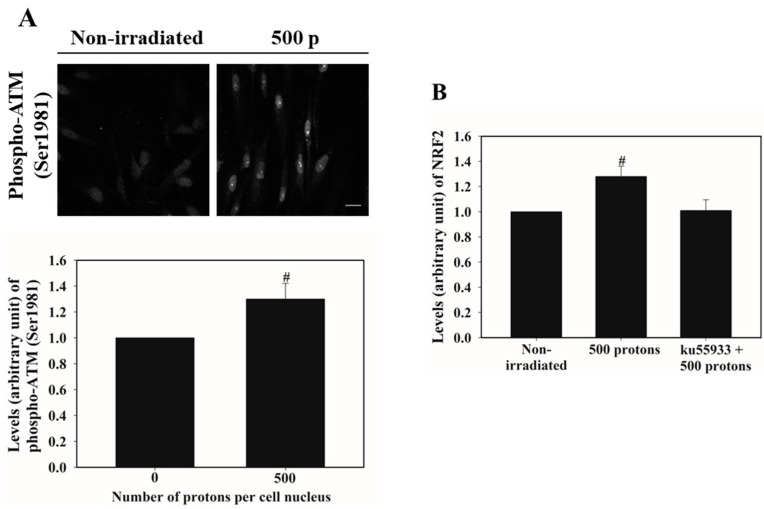
The nucleus of the WI-38 cells was targeted by 500 protons. One hour later, the levels of phosphorylated ATM (Ser1981) were detected by immunofluorescence staining. Scale bar = 30 µm (**A**). After being exposed to 10 µmol/L ku55933 for one hour, WI-38 cells were exposed to 500 proton nucleus irradiation. Then, the cells were incubated for 12 h in the medium containing 10 µmol/L ku55933 (**B**). The nucleus accumulation of NRF2 was detected by immunofluorescence staining. # *p* value < 0.05.

Nucleus targeted irradiation resulted in DSB formation. We then investigated whether the DNA damage response kinase ATM regulated NRF2 nucleus accumulation. Figure 4A shows that 1 h after nucleus irradiation with 500 protons, the levels of phosphorylated ATM at Ser1981 increased by around 30%. Next, we checked the NRF2 nucleus accumulation under the presence of the ATM specific chemical inhibitor ku55933. A final concentration of 10 µmol/L ku55933 was used to pretreat WI-38 cells 1 h prior to 500 proton nucleus irradiation, and the cells were incubated for 12 h after irradiation. Figure 4B showed that ku55933 treatment suppressed NRF2 nucleus accumulation, indicating that ATM activation by nucleus targeted irradiation was partially responsible for NRF2 activation.

### 3.4. ERK 1/2 Activation Promotes NRF2 Nucleus Accumulation

**Figure 5:**
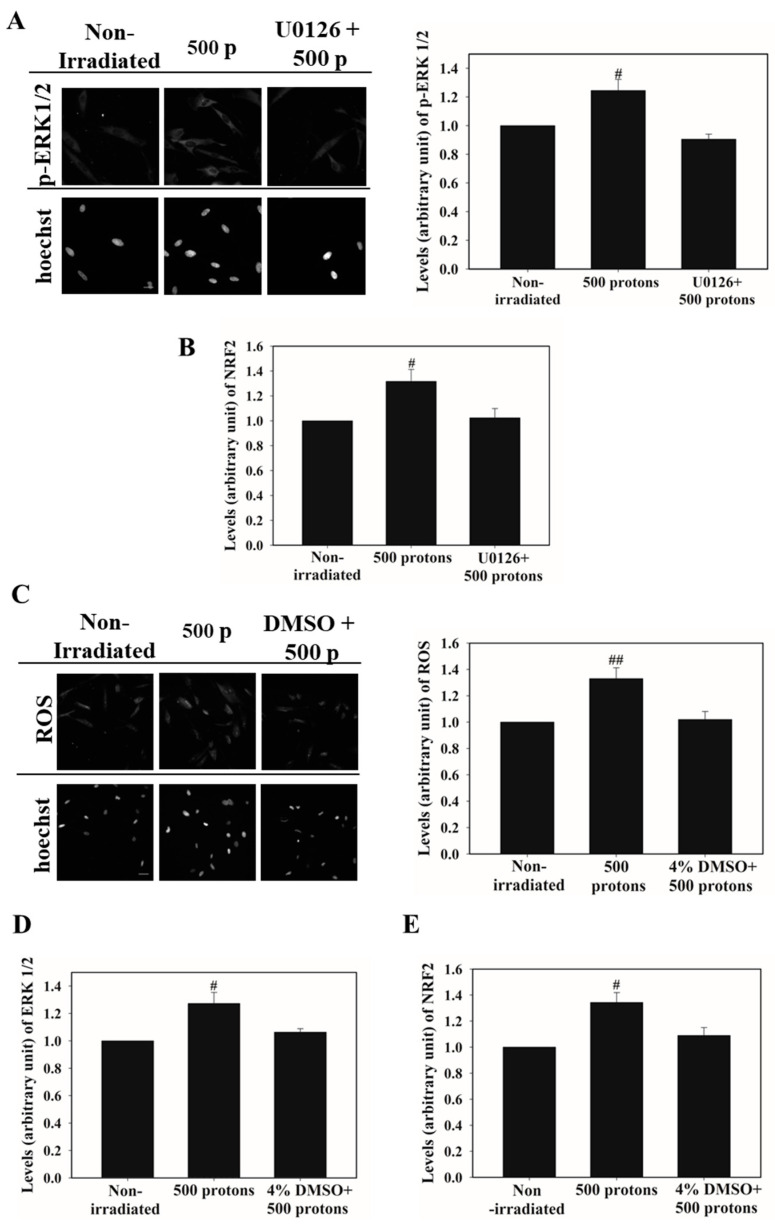
The WI-38 cells were treated or mock treated with 2.5 µmol/L U0126 for 30 min and then exposed to 500 proton nucleus irradiation. Immunofluorescence labeling was used to determine the levels of phosphorylated ERK 1/2 12 h later. Scale bar = 30 µm (**A**), and the WI-38 cells were treated with U0126 and nucleus targeted irradiation according to the conditions described in Figure 5A. Then, the nucleus accumulation of NRF2 was detected by immunofluorescence staining (**B**). Prior to and following 500 proton irradiation, the WI-38 cells were treated with 4% DMSO for 30 min, respectively. Then, the ROS levels were measured with hydroethidine, as described in the “Materials and Methods”. Scale bar = 50 µm (**C**). The WI-38 cells were treated with DMSO and nucleus irradiation using the same conditions in Figure 5C. Immunofluorescence labeling was used to determine the levels of phosphorylated ERK 1/2 (**D**) and nucleus accumulation of NRF2 (**E**) 12 h after irradiation. # *p* value < 0.05. ## *p* value < 0.01.

ERK 1/2 are members of the mitogen-activated protein kinase (MAPK) family and are closely associated with the biological effects of ionizing irradiation. Twelve hours post nucleus targeted irradiation by 500 protons, phospho-ERK 1/2 was detected in the WI-38 cells. Figure 5A shows that when the irradiated cells were compared to the non-irradiated cells, the levels of phospho-ERK 1/2 increased by 25%. We next used a final concentration of 2.5 μmol/L U0126 to inhibit ERK 1/2 (Figure 5A), then detected the nucleus accumulation of NRF2. Figure 5B shows that U0126 treatment clearly suppressed the accumulation of NRF2 caused by targeted irradiation, indicating that the nucleus irradiation activation of ERK 1/2 played an important role in stimulating the nucleus accumulation of NRF2. NRF2 is well-known as an antioxidative response regulator. We then investigated whether ERK 1/2 activation mediated NRF2 nucleus accumulation was linked to ROS production induced by nucleus targeted irradiation. As shown in Figure 5C, the WI-38 cells receiving 500 proton nucleus irradiation displayed around a 35% increase in the level of ROS. The cell was then treated with 4% DMSO for 30 min prior to irradiation and for 10 min afterward to scavenge ROS (Figure 5C). The results of phospho-ERK 1/2 showed that DMSO treatment diminished the activation of ERK 1/2 in the irradiated cells (Figure 5D). Meanwhile, the nucleus accumulation of NRF2 was reduced from around 135% in the irradiated cells to around 110% after DMSO treatment when compared to that in the non-irradiated cells (Figure 5E). These findings suggest that increased ROS production caused by nucleus targeted irradiation can promote NRF2 nucleus accumulation in part by stimulating ERK 1/2 activation.

## 4. Discussion

NRF2 is well-known as a master regulator of oxidative stress in mammalian cells. Cellular detoxification enzymes such as NAD(P)H quinone oxidoreductase 1 (NQO1), HO-1, and sulfiredoxin 1 (SRXN1) are targets of NRF2 [21]. Furthermore, NRF2 was reported to be involved in DNA damage repair, as a glucose metabolism regulator, and inflammation repressor. In irradiated human colonic epithelial cells, Kim et al. [19] discovered that NRF2 bound to antioxidative response elements in the promoter region of 53BP1 to augment DNA damage response, promote DNA damage repair, and remove repairosome. NRF2 bound to the promoter of the DNA repair enzyme OGG1 (8-oxoguanine DNA glycosylase) and stimulated its expression, according to Shang et al. [22]. This finding is of note in reducing drug resistance in the treatment of acute myeloid leukemia. Matsuoka et al. [18] discovered that decreasing NRF2 resulted in decreased glycolysis efficiency, which could be associated with the radio-sensitivity of oral squamous cell carcinoma by monitoring glycolysis and the expression of glycolysis-related genes. Kobayashi et al. [23] demonstrated that NRF2 bound to the proximity of DNA sequences coding for proinflammatory cytokines such as IL-6 and IL-1β, inhibited the transcription and expression of these proinflammation genes, and alleviated inflammation. NRF2 is considered as a potential target in disease treatment or prevention such as cancer radiotherapy and radiation protection.

Microbeam irradiation technology allows researchers to investigate the biological effects induced by specific subcellular region irradiation. Genomic DNA is the main target of radiation-induced genotoxicity. With the discovery that cytoplasmic irradiation caused DNA damages in both targeted cells and non-irradiated bystander cells using microbeam irradiation, the differences in the contribution of the nucleus or cytoplasm targeted irradiation to overall biological effects and how they are regulated have become an important topic. We demonstrated that cytoplasm targeted irradiation stimulated the nucleus accumulation of NRF2 [20]. Using the same cell line, we found that nucleus targeted irradiation stimulated NRF2 nucleus accumulation in this study. Nucleus accumulation of NRF2 increased by 12% 3 h after nucleus targeted irradiation at a dose of 500 protons, but there was no significant increase in cytoplasm targeted cells. In the cytoplasm targeted cells, the nucleus accumulation of NRF2 showed a rising tendency within 24 h post irradiation, whereas in the nucleus targeted cells, the maximal nucleus accumulation of NRF2 was observed 12 h after irradiation. In the dose-dependency study, 100 protons clearly stimulated NRF2 nucleus accumulation in both the cytoplasm targeted and nucleus targeted cells, and 500 proton irradiation further enhanced NRF2 nucleus accumulation. When the proton amount was increased to 1000, the nucleus accumulation of NRF2 in cytoplasm targeted cells continued to rise, while in nucleus targeted cells, the nuclear fraction of NRF2 decreased to a level similar to that in the non-irradiated cells. These reflect the different response of NRF2 signaling to nucleus targeted or cytoplasm targeted irradiation. One possible explanation is that when NRF2 acts as a DNA damage response regulator in nucleus targeted cells, acute DNA damage likely promotes quick NRF2 activation. As reported by Kim et al. [19], NRF2 stimulated the nucleus accumulation of RAD51 and BRCA1 within 2 h after irradiation. Moreover, when compared to cells harboring NRF2 shRNA construct cells, cells harboring the control construct showed a significant decrease in the 53BP1 foci 2 h after irradiation. Jayakumar et al. [24] proposed a ROS independent way of NRF2 in repairing radiation-induced DNA damage. They discovered that NRF2 accelerated the repair of DSB and comet tail in human cancer cells within 2 h after irradiation. In this study, we discovered that ATM was activated in the nucleus targeted cells 1 h after irradiation, and that ATM was an upstream regulator of NRF2 nucleus accumulation. These findings could explain why there was an obvious nucleus accumulation of NRF2 at 3 h after nucleus targeted irradiation. In cytoplasm irradiated cells, the levels of ROS and NO showed sustained elevation. For example, 24 h after cytoplasmic irradiation, the level of NO in the T98G cells was significantly higher than that in the non-irradiated cells [8]. Within 24 h of irradiation, the level of mitochondrial superoxide was significantly higher in the cytoplasm targeted human small airway epithelial cells than in the non-irradiated cells [25]. As a result, long-term elevations of ROS and NO in cytoplasm targeted cells may stimulate NRF2 antioxidative response in a long time range. Direct nucleus irradiation induced more serious DNA damages than direct cytoplasm traversal. Excessive DNA damages, which blocks cellular defensive responses and leads to cell death, may cause a decrease in NRF2 nucleus accumulation at the 1000 proton nucleus targeted cells.

ERK 1/2 are members of the MAPK family, which regulate various cellular processes such as cell division, proliferation, apoptosis, transcription, and translation [26]. ERK 1/2 activation was involved in regulating the radiation induced biological effects. For example, Williams et al. [27] discovered that inhibiting ERK 1/2 activation made pancreatic cancer cells sensitive to radiation. Zhou et al. [28] demonstrated that ERK 1/2 activation in radiation bystander cells was responsible for the occurrence of RIBE by upregulating the expression of cyclo-oxygenase-2 (COX-2). Hong et al. [29] found that in cytoplasm targeted cells, ERK 1/2 were activated to promote the expression of COX-2, and increased genotoxicity. Our group discovered that the activation of ERK 1/2 and NRF2 was involved in the radiation adaptive response induced by cytoplasm targeted irradiation [5]. It was discovered in this study that direct nucleus irradiation induced the activation of ERK 1/2, further stimulating NRF2. These results showed that convergent upstream regulators of NRF2 nucleus accumulation existed in direct nucleus or cytoplasm traversal, even though they may respond to different stimuli. Activation of NRF2 alleviated cytoplasm targeted irradiation induced the DSB levels [20]. Similarly, it reduced the nucleus targeted irradiation-induced DSB levels, indicating NRF2 attenuated both direct and indirect DNA damage. Kim et al. [19] discovered that activating NRF2 in the irradiated cells stimulated the formation of the RAD51 foci, thereby promoting DNA damage repair. In a previous study, cytoplasm targeted irradiation increased the nucleus accumulation of RAD51 and XRCC4, which was boosted by NRF2 pre-activation [20]. Even though we could not find direct evidence that nucleus targeted irradiation stimulated the formation of RAD51 foci in the cell nucleus, previous research has suggested that the NRF2 stimulated nucleus accumulation of DNA damage repair proteins was involved in mitigating both the cytoplasm and nucleus targeted irradiation-induced DNA damage. However, the ROS/NO scavenging role of NRF2 in mitigating cytoplasm or nucleus targeted irradiation-induced DNA damage was not investigated. Thus, whether both “accelerating repair” and “preventing the formation” of DNA damage contributed to attenuate DNA damage in the cytoplasm or nucleus targeted cells deserve further exploration.

## 5. Conclusions

We provide evidence that NRF2 signaling was activated in nucleus targeted human normal fibroblasts by detecting NRF2 nucleus accumulation and the expression of its target gene HO-1 using the SPICE-QST microbeam. The relationships between NRF2 nucleus accumulation and number of protons were investigated as well as the post-irradiation time intervals. The activation of NRF2 alleviated nucleus targeted irradiation-induced the DNA DSB level. Furthermore, we identified that the activated ATM and ERK 1/2 were the upstream regulators of NRF2 nucleus accumulation in the nucleus targeted cells. The findings demonstrate the similarities and differences in the activation of the NRF2 signal in cells exposed to nucleus or cytoplasm irradiation. Together with previous reports, particularly the findings that cytoplasmic irradiation improved DNA damage repair [11], promoted autophagy [30] and glycolysis [31], subcellular targeted irradiation studies aided by microbeam irradiation technology will assist to uncover novel mechanisms on the radiation induced cellular effects.

## Data Availability

Data supporting the findings of this study are available from the corresponding author upon reasonable request.

## References

[1] Prise K.M., Belyakov O.V., Folkard M., Michael B.D. (1998). Studies of bystander effects in human fibroblasts using a charged particle microbeam. Int. J. Radiat. Biol..

[2] Miller R.C., Randers-Pehrson G., Geard C.R., Hall E.J., Brenner D.J. (1999). The oncogenic transforming potential of the passage of single alpha particles through mammalian cell nuclei. Proc. Natl. Acad. Sci. USA.

[3] Autsavapromporn N., Kobayashi A., Liu C., Jaikang C., Tengku Ahmad T.A., Oikawa M., Konishi T. (2022). Hypoxia and proton microbeam: Role of gap junction intercellular communication in inducing bystander responses on human lung cancer cells and normal cells. Radiat. Res..

[4] Kobayashi A., Autsavapromporn N., Ahmad T.A.F.T., Oikawa M., Homma-Takeda S., Furusawa Y., Wang J., Konishi T. (2019). Bystander WI-38 cells modulate DNA double-STRAND break repair in microbeam-targeted A549 cells through gap junction intercellular communication. Radiat. Prot. Dosim..

[5] Wang J., Kobayashi A., Ohsawa D., Oikawa M., Konishi T. (2020). Cytoplasmic radiation induced radio-adaptive response in human lung fibroblast WI-38 cells. Radiat. Res..

[6] Hei T.K., Wu L.J., Liu S.X., Vannais D., Waldren C.A., Randers-Pehrson G. (1997). Mutagenic effects of a single and an exact number of alpha particles in mammalian cells. Proc. Natl. Acad. Sci. USA.

[7] Wu L.J., Randers-Pehrson G., Xu A., Waldren C.A., Geard C.R., Yu Z., Hei T.K. (1999). Targeted cytoplasmic irradiation with alpha particles induces mutations in mammalian cells. Proc. Natl. Acad. Sci. USA.

[8] Shao C., Folkard M., Michael B.D., Prise K.M. (2004). Targeted cytoplasmic irradiation induces bystander responses. Proc. Natl. Acad. Sci. USA.

[9] Konishi T., Oikawa M., Suya N., Ishikawa T., Maeda T., Kobayashi A., Shiomi N., Kodama K., Hamano T., Homma-Takeda S. (2013). SPICE-NIRS microbeam: A focused vertical system for proton irradiation of a single cell for radiobiological research. J. Radiat. Res..

[10] Kobayashi A., Ahmad T.T.A.F., Autsavapromporn N., Oikawa M., Homma-Takeda S., Furusawa Y., Wang J., Konishi T. (2017). Enhanced DNA double-strand break repair of microbeam targeted A549 lung carcinoma cells by adjacent WI38 normal lung fibroblast cells via bi-directional signaling. Mutat. Res..

[11] Konishi T., Kobayashi A., Ahmad T.T.A.F., Wang J. (2018). Enhanced DNA double strand break repair triggered by microbeam irradiation induced cytoplasmic damage. J. Radiat. Cancer Res..

[12] Funayama T. (2019). Heavy-ion microbeams for biological science: Development of system and utilization for biological experiments in QST-Takasaki. Quantum Beam Sci..

[13] Matsumoto Y., Hamada N., Aoki-Nakano M., Funayama T., Sakashita T., Wada S., Kakizaki T., Kobayashi Y., Furusawa Y. (2015). Dependence of the bystander effect for micronucleus formation on dose of heavy-ion radiation in normal human fibroblasts. Radiat. Prot. Dosim..

[14] Autsavapromporn N., Suzuki M., Funayama T., Usami N., Plante I., Yokota Y., Mutou Y., Ikeda H., Kobayashi K., Kobayashi Y. (2013). Gap junction communication and the propagation of bystander effects induced by microbeam irradiation in human fibroblast cultures: The impact of radiation quality. Radiat. Res..

[15] Jella K.K., Moriarty R., McClean B., Byrne H.J., Lyng F.M. (2018). Reactive oxygen species and nitric oxide signaling in bystander cells. PLoS ONE.

[16] Sporn M., Liby K. (2012). NRF2 and cancer: The good, the bad and the importance of context. Nat. Rev. Cancer.

[17] You G., Chang J., Li Y., Huang C., Tsai Y., Fan K., Kang C., Huang S., Chang P., Cheng A. (2022). MYH9 facilitates cell invasion and radioresistance in head and neck cancer via modulation of cellular ROS levels by activating the MAPK-Nrf2-GCLC pathway. Cells.

[18] Matsuoka Y., Yoshida R., Kawahara K., Sakata J., Arita H., Nakashima H., Takahashi N., Hirayama M., Nagata M., Hirosue A. (2022). The antioxidative stress regulator Nrf2 potentiates radioresistance of oral squamous cell carcinoma accompanied with metabolic modulation. Lab. Investig..

[19] Kim S., Pandita R., Eskiocak U., Ly P., Kaisani A., Kumar R., Cornelius C., Wright W., Pandita T., Shay J. (2012). Targeting of Nrf2 induces DNA damage signaling and protects colonic epithelial cells from ionizing radiation. Proc. Natl. Acad. Sci. USA.

[20] Wang J., Konishi T. (2019). Nuclear factor (erythroid-derived 2)-like 2 antioxidative response mitigates cytoplasmic radiation-induced DNA double-strand breaks. Cancer Sci..

[21] Dinkova-Kostova A.T., Copple I.M. (2023). Advances and challenges in therapeutic targeting of NRF2. Trends Pharm. Sci..

[22] Shang Q., Pan C., Zhang X., Yang T., Hu T., Zheng L., Cao S., Feng C., Hu X., Chai X. (2022). Nuclear factor Nrf2 promotes glycosidase OGG1 expression by activating the AKT pathway to enhance leukemia cell resistance to cytarabine. J. Biol. Chem..

[23] Kobayashi E., Suzuki T., Funayama R., Nagashima T., Hayashi M., Sekine H., Tanaka N., Moriguchi T., Motohashi H., Nakayama K. (2016). Nrf2 suppresses macrophage inflammatory response by blocking proinflammatory cytokine transcription. Nat. Commun..

[24] Jayakumar S., Pal D., Sandur S. (2015). Nrf2 facilitates repair of radiation induced DNA damage through homologous recombination repair pathway in a ROS independent manner in cancer cells. Mutat. Res..

[25] Zhang B., Davidson M.M., Zhou H., Wang C., Walker W.F., Hei T.K. (2013). Cytoplasmic irradiation results in mitochondrial dysfunction and DRP1-dependent mitochondrial fission. Cancer Res..

[26] Owens D., Keyse S. (2007). Differential regulation of MAP kinase signalling by dual-specificity protein phosphatases. Oncogene.

[27] Williams T.M., Flecha A.R., Keller P., Ram A., Karnak D., Galbán S., Galbán C.J., Ross B.D., Lawrence T.S., Rehemtulla A. (2012). Cotargeting MAPK and PI3K signaling with concurrent radiotherapy as a strategy for the treatment of pancreatic cancer. Mol. Cancer Ther..

[28] Zhou H., Ivanov V.N., Gillespie J., Geard C.R., Amundson S.A., Brenner D.J., Yu Z., Lieberman H.B., Hei T.K. (2005). Mechanism of radiation-induced bystander effect: Role of the cyclooxygenase-2 signaling pathway. Proc. Natl. Acad. Sci. USA.

[29] Hong M., Xu A., Zhou H., Wu L., Randers-Pehrson G., Santella R.M., Yu Z., Hei T.K. (2010). Mechanism of genotoxicity induced by targeted cytoplasmic irradiation. Br. J. Cancer.

[30] Wu J., Zhang B., Wuu Y.R., Davidson M.M., Hei T.K. (2017). Targeted cytoplasmic irradiation and autophagy. Mutat. Res..

[31] Wu J., Zhang Q., Wuu Y.R., Zou S., Hei T.K. (2017). Cytoplasmic irradiation induces metabolic shift in human small airway epithelial cells via activation of pim-1 kinase. Radiat. Res..

